# Clinical outcomes and safety of preservative-free diclofenac sodium eye drops in SPT-assisted transepithelial photorefractive keratectomy

**DOI:** 10.3389/fphar.2026.1781133

**Published:** 2026-04-15

**Authors:** Huang Zhang, Ming-Ming Li, Shuang Qiu

**Affiliations:** Department of Ophthalmology, Zhongshan Torch Development Zone People’s Hospital, Zhongshan, Guangdong, China

**Keywords:** nonsteroidal anti-inflammatory drug, postoperative pain, pranoprofen, preservative-free diclofenac sodium, smart pulse technology, transepithelial photorefractive keratectomy

## Abstract

**Background:**

Early postoperative pain and epithelial recovery remain important determinants of patient experience after surface ablation refractive surgery. Evidence comparing topical nonsteroidal anti-inflammatory drug (NSAID) options in Smart Pulse Technology (SPT)-assisted transepithelial photorefractive keratectomy (TransPRK) is limited.

**Methods:**

This retrospective cohort study included consecutive patients who underwent SPT-assisted TransPRK between January 2022 and December 2025. Patients received either standard postoperative care plus pranoprofen 0.1% eye drops (control group) or preservative-free diclofenac sodium 0.1% eye drops (PF diclofenac; observation group), administered four times daily for 3 days. Pain was assessed using the visual analog scale (VAS; 0–10) at 2, 24, 48, and 72 h. Secondary outcomes included time to complete epithelialization, time to bandage contact lens (BCL) removal, corneal haze grading, ocular surface and inflammatory signs, and adverse events.

**Results:**

A total of 239 patients were analyzed (control, n = 118; observation, n = 121). The observation group had significantly lower VAS pain scores at 2 h (5.40 ± 1.70 vs. 4.80 ± 1.60; P = 0.005), 24 h (4.20 ± 1.60 vs. 3.40 ± 1.50; P < 0.001), 48 h (2.80 ± 1.30 vs. 2.10 ± 1.20; P < 0.001), and 72 h (1.60 ± 1.00 vs. 1.20 ± 0.90; P = 0.001). A higher proportion of patients in the observation group achieved mild pain (VAS ≤ 3) at 24 h (53.7% vs. 38.1%; P = 0.016). Time to complete epithelialization (4.05 ± 0.88 vs. 4.32 ± 0.92 days; P = 0.021) and BCL removal (4.25 ± 0.90 vs. 4.56 ± 0.98 days; P = 0.012) were shorter in the observation group. Corneal haze and adverse events were comparable between groups, and no serious adverse events were observed.

**Conclusion:**

In SPT-assisted TransPRK, PF diclofenac was associated with improved early pain control and modestly faster epithelial recovery, without detectable differences in haze or short-term safety compared with pranoprofen.

## 1 Introduction

Photorefractive keratectomy (PRK) and contemporary transepithelial PRK (TransPRK) remain widely used surface-ablation options for refractive error correction, particularly in candidates in whom flap-based procedures may be less desirable. Recent technical refinements have focused on improving ablation regularity, epithelial removal efficiency, and early optical quality. In this context, Smart Pulse Technology (SPT)–enabled TransPRK has been incorporated into several commercial platforms and is intended to optimize stromal surface smoothness and thereby support predictable refractive and visual outcomes across a range of myopic severities (Margarit et al., 2025; Akram et al., 2025). Recent clinical evidence and systematic evaluations indicate that single-step TransPRK can achieve favorable short- to mid-term efficacy, safety, and predictability, while corneal epithelial remodeling after TransPRK may be objectively characterized by optical coherence tomography–derived epithelial thickness metrics (Sh et al., 2025; Weng et al., 2024).

Despite advances in postoperative care, early pain remains a significant challenge after surface ablation. Pain is primarily caused by epithelial defects, exposure of corneal nociceptors, and inflammatory mediators in the initial postoperative days. Current strategies, including bandage contact lenses, topical anesthetics, and nonsteroidal anti-inflammatory drugs (NSAIDs), aim to manage pain while promoting epithelial healing. Recent studies have demonstrated the effectiveness of preservative-free (PF) topical NSAIDs, such as diclofenac, for pain relief after PRK without significant delays in epithelial recovery. However, concerns regarding epithelial intolerance with certain formulations highlight the importance of evaluating PF NSAIDs for safer postoperative management in corneal surface ablation (Abdel-Radi et al., 2023; Gomel et al., 2024). A key gap in the existing literature is the lack of direct comparison between PF diclofenac sodium and pranoprofen specifically in the context of SPT-assisted TransPRK (Fukuda et al., 2024; Hafezi et al., 2022). While various studies have explored the efficacy of different NSAIDs in managing postoperative pain following refractive surgeries such as PRK, the unique combination of TransPRK, SPT technology, and specific NSAID regimens has not been thoroughly investigated (Kahook et al., 2024; Sjö et al., 2025; Zarei-Ghanavati et al., 2025). Additionally, most studies have not focused on both pain management and epithelial healing together in this specific patient group (Hafezi et al., 2022; Ho, 2024).

In light of this gap, our study aims to investigate whether PF diclofenac sodium provides superior postoperative pain control and more rapid epithelial recovery compared to pranoprofen in SPT-assisted TransPRK patients. By comparing these two NSAIDs directly within this specific surgical context, we seek to address this knowledge gap, offering potential insights into optimizing postoperative care for TransPRK patients, especially with respect to minimizing pain and improving healing outcomes.

## 2 Methods

### 2.1 Study design

This single-center retrospective cohort study consecutively enrolled patients who underwent SPT-assisted transepithelial photorefractive keratectomy (TransPRK) at our institution between January 2022 and December 2025. Only one eye per patient was included in the analysis. According to the postoperative anti-inflammatory/analgesic regimen recorded in the medical charts, patients were categorized into a control group receiving standard postoperative care in combination with topical pranoprofen eye drops and an observation group receiving preservative-free diclofenac sodium (PF diclofenac) eye drops as the topical NSAID component of postoperative management. Eligible patients were required to (i) have a definitive indication for primary refractive surgery with preoperative examinations supporting suitability for TransPRK (including stable refraction and adequate corneal status), (ii) complete perioperative documentation (preoperative assessment, intraoperative parameters, and postoperative medication records), and (iii) available postoperative follow-up sufficient for outcome assessment. Exclusion criteria included any prior ocular surgery; suspected corneal ectatic disorders or topographic/tomographic abnormalities precluding surface ablation; active ocular infection or inflammation at baseline; clinically significant ocular surface disease likely to confound epithelial healing; severe systemic conditions affecting wound healing or concurrent immunosuppressive therapy; pregnancy or lactation; documented hypersensitivity to diclofenac, pranoprofen, aspirin/other NSAIDs, or relevant excipients; and incomplete records or loss to follow-up preventing ascertainment of key endpoints. The study was reviewed and approved by the hospital’s ethics committee and conducted in accordance with relevant guidelines and the Declaration of Helsinki. All data were handled confidentially, with personal identifiers removed prior to analysis to ensure participant privacy.

### 2.2 Surgical technique

All SPT-assisted TransPRK procedures were performed using a standardized protocol. Following topical anesthesia and sterile preparation, an eyelid speculum was placed and treatment centration was achieved according to the routine alignment strategy of the excimer laser system with active eye tracking. The procedure was delivered in a single-step transepithelial sequence, consisting of excimer laser–mediated epithelial removal using a predefined epithelial profile, followed immediately by stromal refractive ablation based on the preoperative treatment plan. After completion of ablation, the corneal surface was thoroughly irrigated with sterile balanced salt solution, and a therapeutic bandage soft contact lens was applied. The bandage lens was removed after slit-lamp examination with fluorescein staining confirmed complete epithelialization, defined as the absence of an epithelial defect. To minimize procedural variability and performance bias, all surgeries were performed by the same experienced surgeon throughout the study period.

### 2.3 Group allocation and postoperative interventions

Patients were allocated in a non-randomized manner according to the postoperative NSAID regimen documented in the electronic medical record. The control group received conventional postoperative care plus topical pranoprofen 0.1% eye drops (Pranoprofen Eye Drops, Guangdong Zhongsheng Pharmaceutical Co., Ltd., Dongguan, China) one drop four times daily for 3 days after surgery, whereas the observation group received the same conventional postoperative care plus commercially available PF diclofenac 0.1% eye drops (Diclofenac Sodium Eye Drops [Difei], Shenyang Xingqi Pharmaceutical Co., Ltd., Shenyang, China) one drop four times daily for 3 days. Conventional postoperative care was standardized across both groups and included: (1) a topical fluoroquinolone antibiotic (levofloxacin 0.5%, one drop four times daily) during the immediate postoperative period and continued until corneal re-epithelialization and/or bandage contact lens (BCL) removal; (2) topical anti-inflammatory therapy initiated only after complete epithelial closure and/or BCL removal had been confirmed, with an identical initiation timing and tapering schedule in both groups, consisting of a steroid–antibiotic combination (tobramycin–dexamethasone, one drop four times daily for 2 weeks), followed by a topical corticosteroid (fluorometholone 0.1%, one drop four times daily for 2 weeks), with subsequent tapering performed according to the same protocol in both groups; and (3) PF artificial tears administered frequently (e.g., every 2 h while awake) until complete epithelial healing, then reduced to a maintenance schedule (e.g., four times daily) for several months to support ocular-surface recovery. A BCL was placed at the end of surgery in all eyes and removed when complete corneal re-epithelialization was confirmed on slit-lamp examination.

Postoperative follow-up was performed according to a predefined schedule (typically at postoperative day 1, day 3, and week 1, followed by visits at 1, 3, 6, and 12 months), with additional interim assessments arranged when epithelial healing was delayed or when patients reported disproportionate discomfort. To reduce treatment contamination, concomitant medication restrictions were applied throughout the early postoperative period: additional topical or systemic analgesics (beyond the protocol), other NSAIDs, and non-protocol topical agents were not permitted.

### 2.4 Data collection and data quality control

Data were obtained retrospectively from the institutional electronic medical record system and the refractive surgery database. Eligible cases were identified using operative logs for SPT-assisted TransPRK within the prespecified study period, and patient identifiers were cross-checked against outpatient and follow-up records to confirm postoperative NSAID exposure and outcome availability. A standardized data extraction form was used to collect variables in three domains: (1) baseline characteristics, including age, sex, preoperative manifest refraction (sphere, cylinder, and spherical equivalent), and preoperative visual acuity (UDVA and CDVA); (2) corneal and surgical parameters, including corneal pachymetry and keratometry, planned optical/transition zone settings, ablation depth and/or laser ablation time where recorded, and intraoperative events; and (3) postoperative course and outcomes, including pain scores at 2 h, 24 h (postoperative day 1), 48 h (postoperative day 2), and 72 h (postoperative day 3) after surgery, time to complete epithelialization confirmed by slit-lamp examination with fluorescein staining, bandage contact lens removal time, haze grading at follow-up visits, ocular surface and anterior segment inflammatory signs, and adverse events and management actions (e.g., rescue medications, additional treatment, or discontinuation of the assigned NSAID). Medication exposure (agent, dosing frequency, duration, and protocol deviations) was abstracted from prescription records and clinician documentation. Preoperative dry-eye risk/meibomian gland dysfunction (MGD) status was assessed from routine clinical records obtained before surgery, including slit-lamp examination findings and ocular-surface evaluation documented in the refractive surgery workup. MGD severity was categorized according to the treating clinician’s preoperative assessment of lid-margin abnormalities, meibum expressibility, and secretion quality as documented in the medical chart. Based on these preoperative findings, patients were classified as having low risk with none-to-mild MGD or high risk with moderate-to-severe MGD. To ensure data reliability, two investigators independently extracted key outcomes and exposure variables, and discrepancies were resolved by consensus with adjudication by a senior clinician when necessary. Logical range checks and consistency checks were performed (verification of postoperative time stamps, plausibility of refractive and pachymetry values, and alignment between slit-lamp notes and the recorded timing of epithelial closure/BCL removal). Missing data were documented for each variable; cases lacking essential exposure information or primary outcome ascertainment were excluded according to predefined criteria.

### 2.5 Statistical analysis

All statistical analyses were performed using IBM SPSS Statistics, version 26.0 (IBM Corp., Armonk, NY, United States). Continuous variables were examined for distributional characteristics and are presented as mean ± standard deviation for approximately normally distributed data or as median (interquartile range) for non-normally distributed data. Categorical variables are summarized as number (percentage). Between-group comparisons were selected according to variable type and distribution. For continuous variables, independent-samples t tests were used for normally distributed data; when variance heterogeneity was detected, the Welch t-test was applied. For non-normally distributed continuous or ordinal outcomes, the Mann–Whitney U test was used. Categorical variables were compared using the chi-square test, and Fisher’s exact test was applied when expected cell counts were small. Pain scores were compared between groups at each predefined time point (2, 24, 48, and 72 h). Ordinal corneal haze grades were compared between groups using nonparametric methods. Effect sizes were reported to quantify between-group differences, including standardized mean differences for continuous outcomes and odds ratios with 95% confidence intervals for categorical outcomes; risk differences with 95% confidence intervals were additionally provided for selected binary endpoints. Prespecified subgroup analyses were conducted by stratifying patients according to preoperative myopia severity (based on spherical equivalent) and preoperative dry-eye risk/MGD status, classified as low risk with none-to-mild MGD or high risk with moderate-to-severe MGD, with the same between-group methods applied within strata. Sensitivity analyses included a per-protocol analysis excluding patients with documented deviations from the assigned NSAID regimen during the early postoperative period, nonparametric re-analyses of key outcomes, and multivariable linear regression models adjusting for prespecified baseline covariates. Before fitting the multivariable models, multicollinearity among covariates was assessed using tolerance statistics and variance inflation factors (VIFs). A VIF < 5 was considered to indicate the absence of problematic multicollinearity. All tests were two-sided, and a P value < 0.05 was considered statistically significant.

## 3 Results

### 3.1 Patient selection and study population

A total of 267 consecutive patients who underwent SPT-assisted TransPRK during the study period were identified from the institutional surgical database and screened for eligibility. Twenty-eight patients were excluded after record review for the following reasons: not meeting the prespecified inclusion criteria (n = 9), pre-existing clinically relevant ocular surface disease (n = 7), history of prior ocular surgery (n = 5), incomplete clinical records precluding ascertainment of key variables/endpoints (n = 4), and insufficient follow-up or early loss to follow-up within the minimum observation window (n = 3). The remaining 239 patients were included in the final analysis, comprising 118 patients in the control group (routine postoperative care plus pranoprofen) and 121 patients in the observation group (routine postoperative care plus PF diclofenac).

### 3.2 Baseline and perioperative comparability

Baseline demographic characteristics, preoperative refractive status, corneal parameters, and visual function were comparable between the control (pranoprofen) and observation (PF diclofenac) groups (Table 1). No statistically significant between-group differences were observed for age (28.38 ± 5.62 vs. 27.64 ± 5.12 years; P = 0.289) or sex distribution (male, 59.3% vs. 55.4%; P = 0.537). Preoperative refractive indices, including spherical refraction, cylindrical refraction, and spherical equivalent, did not differ between groups (all P ≥ 0.780). Likewise, corneal structural and curvature parameters (central corneal thickness, Kmean, Kmax, and corneal astigmatism) and preoperative visual acuity (UDVA and CDVA, logMAR) were balanced (all P ≥ 0.471). Perioperative and surgical parameters were also similar across groups, including optical/transition zone settings, ablation depth, laser ablation time, and total operative time (all P ≥ 0.619). Intraoperative events were rare, with suction loss documented in 1 (0.8%) and 2 (1.7%) patients in the control and observation groups, respectively, and no clinically significant decentration recorded in either group. The duration of bandage contact lens wear did not differ significantly (4.10 ± 0.90 vs. 4.00 ± 0.80 days; P = 0.365). Standardized perioperative co-interventions (topical antibiotic prophylaxis, topical corticosteroid therapy, PF artificial tears, and bandage contact lens application) were implemented in all patients. Documented deviation from the assigned NSAID dosing schedule within the first 3 postoperative days was infrequent and comparable between groups (6.8% vs. 8.3%; P = 0.664). Overall, effect sizes were small (absolute SMD ≤ 0.14), supporting baseline and perioperative balance.

**TABLE 1:** Baseline demographic, refractive, corneal, and visual characteristics.

Variable	Control (pranoprofen) (n = 118)	Observation (PF diclofenac) (n = 121)	Test statistic	P value	SMD
Demographics					
Age, years	28.38 ± 5.62	27.64 ± 5.12	t = 1.06	0.289	0.14
Male sex, n (%)	70 (59.3)	67 (55.4)	χ^2^ = 0.38	0.537	0.08
Preoperative refractive status					
Spherical refraction, D	−4.38 ± 1.82	−4.37 ± 1.66	t = −0.04	0.965	−0.01
Cylindrical refraction, D	−0.86 ± 0.55	−0.84 ± 0.53	t = −0.28	0.780	−0.04
Spherical equivalent (SE), D	−4.81 ± 1.85	−4.78 ± 1.66	t = −0.13	0.899	−0.02
Corneal parameters					
Central corneal thickness, µm	536.99 ± 26.04	538.38 ± 28.04	t = −0.40	0.692	−0.05
Mean keratometry (Kmean), D	43.76 ± 1.31	43.88 ± 1.26	t = −0.72	0.471	−0.09
Maximum keratometry (Kmax), D	44.92 ± 1.53	44.89 ± 1.52	t = 0.15	0.879	0.02
Corneal astigmatism, D	0.97 ± 0.52	0.93 ± 0.48	t = 0.62	0.537	0.08
Preoperative visual function					
Preoperative UDVA, logMAR	1.00 ± 0.22	1.00 ± 0.20	t = 0.00	1.000	0.00
Preoperative CDVA, logMAR	0.02 ± 0.04	0.02 ± 0.04	t = −0.39	0.700	−0.05
Perioperative and surgical parameters					
Optical zone, mm	6.35 ± 0.20	6.34 ± 0.22	t = 0.37	0.713	0.05
Transition zone, mm	1.20 ± 0.15	1.21 ± 0.16	t = −0.50	0.619	−0.06
Ablation depth, µm	72.50 ± 14.80	73.10 ± 15.20	t = −0.31	0.757	−0.04
Laser ablation time, s	36.80 ± 6.40	37.10 ± 6.10	t = −0.37	0.711	−0.05
Total operative time, min	8.90 ± 1.60	8.80 ± 1.50	t = 0.50	0.619	0.06
Suction loss, n (%)	1 (0.8)	2 (1.7)	Fisher	1.000	−0.07
Clinically significant decentration, n (%)	0 (0.0)	0 (0.0)	Fisher	1.000	0.00
Bandage contact lens wear, days	4.10 ± 0.90	4.00 ± 0.80	t = 0.91	0.365	0.12
Any deviation from assigned NSAID dosing (first 3 days), n (%)	8 (6.8)	10 (8.3)	χ^2^ = 0.19	0.664	−0.06

CDVA, corrected distance visual acuity; D, diopter; Kmax, maximum keratometry; Kmean, mean keratometry; logMAR, logarithm of the minimum angle of resolution; NSAID, nonsteroidal anti-inflammatory drug; PF, preservative-free; SE, spherical equivalent; SMD, standardized mean difference; UDVA, uncorrected distance visual acuity.

### 3.3 Postoperative pain control

Postoperative pain decreased progressively during the first 72 h after surgery in both groups; however, patients receiving PF diclofenac consistently had lower VAS pain scores than those receiving pranoprofen at 2, 24, 48, and 72 h (all P ≤ 0.005; Table 2). The magnitude of the between-group difference was greatest at 24–48 h, with small-to-moderate effect sizes. The observation group also had a significantly higher proportion of patients with mild pain (VAS ≤ 3) at 24 h, while differences at later time points remained directionally favorable but were not statistically significant. Rescue analgesia was infrequent and did not differ significantly between groups.

**TABLE 2:** Postoperative pain outcomes and clinically meaningful endpoints within 72 h.

Outcome	Control (pranoprofen)	Observation (PF diclofenac)	Mean difference/Effect	Test statistic	P value	Effect size
VAS pain score (0–10), 2 h	5.40 ± 1.70; 5 [4–7]	4.80 ± 1.60; 5 [4–6]	MD = 0.60	t = 2.81	0.005	SMD = 0.36
VAS pain score (0–10), 24 h	4.20 ± 1.60; 4 [3–5]	3.40 ± 1.50; 3 [2–4]	MD = 0.80	t = 3.99	<0.001	SMD = 0.52
VAS pain score (0–10), 48 h	2.80 ± 1.30; 3 [2–4]	2.10 ± 1.20; 2 [1–3]	MD = 0.70	t = 4.32	<0.001	SMD = 0.56
VAS pain score (0–10), 72 h	1.60 ± 1.00; 1 [1–2]	1.20 ± 0.90; 1 [0–2]	MD = 0.40	t = 3.25	0.001	SMD = 0.42
VAS ≤ 3 at 24 h, n (%)	45 (38.1)	65 (53.7)	—	χ^2^ = 5.84	0.016	OR = 1.88 (95% CI 1.12–3.15)
VAS ≤ 3 at 48 h, n (%)	82 (69.5)	97 (80.2)	—	χ^2^ = 3.62	0.057	OR = 1.77 (95% CI 0.98–3.21)
VAS ≤ 3 at 72 h, n (%)	104 (88.1)	115 (95.0)	—	χ^2^ = 3.72	0.054	OR = 2.58 (95% CI 0.96–6.96)
Rescue analgesia within 72 h, n (%)	7 (5.9)	3 (2.5)	—	Fisher’s exact	0.212	OR = 0.40 (95% CI 0.10–1.60)

CI, confidence interval; MD, mean difference (Control − Observation); OR, odds ratio (Observation vs. Control); PF, preservative-free; SMD, standardized mean difference; VAS, visual analog scale.

### 3.4 Epithelial healing and bandage contact lens removal

The observation group treated with PF diclofenac demonstrated a shorter time to complete epithelialization compared with the control group (4.05 ± 0.88 vs. 4.32 ± 0.92 days; t = 2.32, P = 0.021), corresponding to a small effect size (SMD = 0.30). Consistently, the time to BCL removal was also shorter in the observation group (4.25 ± 0.90 vs. 4.56 ± 0.98 days; t = 2.55, P = 0.012; SMD = 0.33). Regarding clinically relevant delays in epithelial recovery, delayed epithelialization (>5 days) occurred in 12 of 118 patients (10.2%) in the control group and 6 of 121 patients (5.0%) in the observation group. Although the point estimates favored PF diclofenac (OR = 0.46, 95% CI 0.17–1.27; RD = −5.2%, 95% CI −11.9% to 1.5%), the between-group difference did not reach statistical significance (Fisher’s exact P = 0.147) (Table 3).

**TABLE 3:** Corneal epithelial healing and bandage contact lens (BCL) removal outcomes.

Outcome	Control (pranoprofen) (n = 118)	Observation (PF diclofenac) (n = 121)	Between-group comparison	P value	Effect size
Time to complete epithelialization, days	4.32 ± 0.92; 4.0 [4.0–5.0]	4.05 ± 0.88; 4.0 [3.0–4.0]	t = 2.32 (Welch)	0.021	SMD = 0.30
Time to BCL removal, days	4.56 ± 0.98; 5.0 [4.0–5.0]	4.25 ± 0.90; 4.0 [4.0–5.0]	t = 2.55 (Welch)	0.012	SMD = 0.33
Delayed epithelialization (>5 days), n (%)	12 (10.2)	6 (5.0)	Fisher’s exact	0.147	OR = 0.46 (95% CI 0.17–1.27); RD = −5.2% (95% CI −11.9% to 1.5%)

BCL, bandage contact lens; CI, confidence interval; OR, odds ratio (Observation vs. Control); PF, preservative-free; RD, risk difference (Observation − Control); SMD, standardized mean difference.

### 3.5 Corneal haze and inflammatory signs

No significant between-group differences were observed in corneal haze severity at 1, 3, or 6 months (all P > 0.05; Table 4). Moderate-to-severe haze was infrequent throughout follow-up and remained comparable between groups. Regarding postoperative inflammatory signs, corneal fluorescein staining on day 3 was significantly lower in the observation group, whereas the proportion of eyes with higher-grade staining did not differ significantly. Conjunctival hyperemia on day 1 and anterior chamber cells were also comparable between groups, indicating no detectable difference in early anterior segment inflammatory response apart from less epithelial staining in the PF diclofenac group.

**TABLE 4:** Corneal haze and postoperative inflammatory signs.

Outcome	Control (pranoprofen)	Observation (PF diclofenac)	Test statistic	P value	Effect size/Notes
Corneal haze grade (Fantes), 1 month	0: 94 (79.7); 1: 22 (18.6); ≥2: 2 (1.7)	0: 103 (85.1); 1: 17 (14.0); ≥2: 1 (0.8)	U = 7535.0	0.262	OR (≥2, Obs vs. Ctrl) 0.48 (95% CI 0.04–5.40); Fisher P = 0.619
Corneal haze grade (Fantes), 3 months	0: 84 (71.2); 1: 29 (24.6); ≥2: 5 (4.2)	0: 94 (77.7); 1: 24 (19.8); ≥2: 3 (2.5)	U = 7619.5	0.237	OR (≥2, Obs vs. Ctrl) 0.57 (95% CI 0.13–2.46); Fisher P = 0.496
Corneal haze grade (Fantes), 6 months	0: 104 (88.1); 1: 13 (11.0); ≥2: 1 (0.8)	0: 110 (90.9); 1: 10 (8.3); ≥2: 1 (0.8)	U = 7335.5	0.489	OR (≥2, Obs vs. Ctrl) 0.97 (95% CI 0.06–15.77); Fisher P = 1.000
Corneal fluorescein staining (Oxford 0–5), day 3	1.75 ± 0.83; 2 [1–2]	1.49 ± 0.82; 1 [1–2]	U = 8415.0	0.010	—
Staining grade ≥3, day 3, n (%)	18 (15.3)	11 (9.1)	χ^2^ = 2.13	0.145	OR (Obs vs. Ctrl) 0.56 (95% CI 0.25–1.23)
Conjunctival hyperemia grade (0–3), day 1	1.07 ± 0.66; 1 [1–1]	0.98 ± 0.67; 1 [1–1]	U = 7616.0	0.307	—
Anterior chamber cells ≥1+ (SUN), day 1, n (%)	1 (0.8)	1 (0.8)	Fisher	1.000	OR (Obs vs. Ctrl) 0.97 (95% CI 0.06–15.77)

CI, confidence interval; OR, odds ratio; PF, preservative-free; SUN, standardization of uveitis nomenclature; U, Mann–Whitney U.

Grading definitions: Fantes haze grade (0–4; higher indicates greater haze); Oxford fluorescein staining (0–5; higher indicates more epithelial staining); conjunctival hyperemia graded 0–3 (higher indicates more hyperemia).

### 3.6 Safety outcomes: adverse events and complications

At the patient level, the overall incidence of adverse events was 11.9% (14/118) in the control group and 7.4% (9/121) in the observation group, without a statistically significant difference (Fisher P = 0.278; OR 0.60, 95% CI 0.25–1.44). A descriptive eye-level summary showed a similar pattern (7.9% vs. 5.1%; Fisher P = 0.260). Key events were infrequent. Suspected infectious keratitis occurred in 2 patients (1.7%) in the control group and 1 patient (0.8%) in the observation group (Fisher P = 0.619), and no confirmed infectious keratitis was recorded in either group. No cases of corneal melt were observed. Medication-related epithelial toxicity was reported in 4.2% vs. 2.5% of patients (Fisher P = 0.496). Loss of ≥2 lines of CDVA at postoperative month 6 was rare (0.8% in each group; Fisher P = 1.000). Persistent pain beyond 72 h requiring an unscheduled visit and allergic reactions were uncommon and did not differ between groups (all P > 0.05). Additional treatment or NSAID discontinuation was required in 5.1% vs. 3.3% of patients (Fisher P = 0.536). No serious adverse events (SAE) occurred in either group (Table 5).

**TABLE 5:** Adverse events and complications through postoperative month 6.

Outcome	Control (pranoprofen) n/N (%)	Observation (PF diclofenac) n/N (%)	Test statistic	P value	OR (95% CI)
Any adverse event (patient-level)	14/118 (11.9)	9/121 (7.4)	Fisher	0.278	0.60 (0.25–1.44)
Any ocular adverse event (eye-level)	18/228 (7.9)	12/234 (5.1)	Fisher	0.260	0.63 (0.30–1.34)
Suspected infectious keratitis	2/118 (1.7)	1/121 (0.8)	Fisher	0.619	0.48 (0.04–5.40)
Medication-related epithelial toxicity	5/118 (4.2)	3/121 (2.5)	Fisher	0.496	0.57 (0.13–2.46)
Loss of ≥2 lines CDVA at month 6	1/118 (0.8)	1/121 (0.8)	Fisher	1.000	0.98 (0.06–15.77)
Persistent pain >72 h requiring unscheduled visit	4/118 (3.4)	2/121 (1.7)	Fisher	0.442	0.48 (0.09–2.67)
Allergic reaction (periocular/ocular)	2/118 (1.7)	1/121 (0.8)	Fisher	0.619	0.48 (0.04–5.40)
Additional treatment or NSAID discontinuation	6/118 (5.1)	4/121 (3.3)	Fisher	0.536	0.64 (0.18–2.32)

CDVA, corrected distance visual acuity; CI, confidence interval; NSAID, nonsteroidal anti-inflammatory drug; PF, preservative-free; OR, odds ratio; SAE, serious adverse event.

### 3.7 Subgroup analysis of 24-h postoperative pain

As shown in Table 6, the observation group consistently exhibited lower 24-h postoperative pain scores than the control group across all prespecified subgroups. This pattern was observed regardless of preoperative myopia severity and was also maintained across strata defined by preoperative dry-eye risk/MGD status. These findings suggest that the early analgesic advantage associated with PF diclofenac was broadly consistent across different baseline refractive and ocular-surface profiles.

**TABLE 6:** Subgroup analysis of 24-h postoperative pain according to preoperative myopia severity and dry-eye risk/meibomian gland dysfunction (MGD) status.

Stratification	Subgroup (definition)	n (Control)	n (Observation)	Pain at 24 h (VAS 0–10), mean ± SD (Control vs. Obs)	MD (C−O)	t	P value
Preoperative myopia (by SE)	Low (SE > −3.00 D)	38	40	4.00 ± 1.50 vs. 3.20 ± 1.40	0.80	2.43	0.017
	Moderate (−6.00 to −3.00 D)	55	56	4.20 ± 1.60 vs. 3.40 ± 1.50	0.80	2.72	0.008
	High (SE ≤ −6.00 D)	25	25	4.60 ± 1.70 vs. 3.50 ± 1.60	1.10	2.36	0.023
Preoperative dry-eye risk/MGD status	Low risk/none–mild MGD	74	76	4.10 ± 1.55 vs. 3.30 ± 1.45	0.80	3.26	0.001
	High risk/moderate–severe MGD	44	45	4.45 ± 1.65 vs. 3.55 ± 1.55	0.90	2.65	0.010

C−O, mean difference (Control − Observation); MGD, meibomian gland dysfunction; Obs, observation group (preservative-free diclofenac); SE, spherical equivalent; VAS, visual analog scale.

Preoperative dry-eye risk/MGD, status was classified from chart-documented ocular-surface assessment as low risk with none-to-mild MGD or high risk with moderate-to-severe MGD.

### 3.8 Sensitivity analyses

Sensitivity analyses were performed to assess the robustness of the primary efficacy findings for 24-h postoperative pain and time to complete epithelialization (Table 7). The between-group differences remained directionally and statistically consistent in the per-protocol analysis, in nonparametric re-analysis, and after multivariable adjustment for prespecified baseline covariates. Overall, these analyses supported the robustness of the main findings, indicating that PF diclofenac remained associated with lower early postoperative pain and shorter epithelial healing time across alternative analytic approaches. Collinearity diagnostics showed no evidence of problematic multicollinearity among the covariates included in the adjusted models, with all VIF values below 5 (Supplementary Table S1).

**TABLE 7:** Sensitivity analyses for primary efficacy endpoints.

Analysis approach	n (Control)	n (Observation)	24-h pain (VAS 0–10), mean ± SD (Control vs. Obs)	MD (C−O)	Test statistic	P value	Time to complete epithelialization, days (Control vs. Obs)	MD (C−O)	Test statistic	P value
Primary analysis (as analyzed)	118	121	4.20 ± 1.60 vs. 3.40 ± 1.50	0.8	t = 3.99	<0.001	4.32 ± 0.92 vs. 4.05 ± 0.88	0.27	t = 2.32	0.021
Per-protocol*	110	111	4.18 ± 1.58 vs. 3.36 ± 1.47	0.82	t = 3.99	<0.001	4.30 ± 0.90 vs. 4.02 ± 0.86	0.28	t = 2.36	0.019
Alternative test (nonparametric)^†^	118	121	Median 4 [3–5] vs. 3 [2–4]	—	U = 5710.0	<0.001	Median 4.0 [4.0–5.0] vs. 4.0 [3.0–4.0]	—	U = 6230.0	0.02
Multivariable-adjusted model^‡^	118	121	Adjusted β (Obs vs. Control) = −0.79	—	t = −4.02	<0.001	Adjusted β (Obs vs. Control) = −0.26	—	t = −2.31	0.022

* Per-protocol set excludes patients with any documented deviation from assigned NSAID, dosing during the first 3 postoperative days (Control: 8; Observation: 10).

^†^ Mann–Whitney U test; medians and interquartile ranges are shown for interpretability; U is reported for the between-group comparison.

^‡^ Linear regression with group as the main exposure and adjustment for prespecified baseline covariates (age, sex, preoperative SE, central corneal thickness, mean keratometry, preoperative dry-eye risk/MGD status, and ablation depth). β represents the adjusted mean difference (Observation − Control).

### 3.9 Post hoc power analysis

Post hoc power analysis was conducted for the two primary outcomes using a two-tailed test with α = 0.05. Continuous variables were analyzed using the independent samples t-test, and Cohen’s d was calculated to assess the effect size. The combined weighted *post hoc* power for the two primary outcomes, 24-h pain and time to complete epithelialization, was 80.5% (>80%), indicating that the current sample size is sufficient to support the detection of the expected effects.

## 4 Discussion

This study provides valuable insights into the efficacy of PF diclofenac in comparison to pranoprofen for postoperative pain management following SPT-assisted TransPRK. The findings indicate that PF diclofenac significantly reduced 24-h postoperative pain and expedited epithelial healing compared to pranoprofen, making it a promising alternative for improving early postoperative outcomes in patients undergoing refractive surgery (Steigleman et al., 2023). These results are noteworthy given the growing interest in optimizing postoperative recovery while minimizing the risk of complications associated with commonly used NSAIDs. The novelty of this study lies in its focus on comparing two widely used NSAIDs with a focus on their postoperative analgesic efficacy and healing outcomes in the context of TransPRK, a technique that is gaining popularity due to its potential to minimize discomfort and enhance recovery time. The study design, leveraging a retrospective cohort and real-world data, is particularly valuable for assessing the practical clinical benefits of PF diclofenac. By focusing on objective, clinically meaningful outcomes such as pain reduction and epithelial healing, this research provides strong evidence for a more personalized approach to postoperative care. Clinically, these findings suggest that PF diclofenac could offer an effective and well-tolerated alternative for managing postoperative pain, potentially enhancing the overall patient experience and expediting recovery. The reduction in epithelial healing time observed with PF diclofenac aligns with the need for therapies that accelerate recovery, thereby minimizing the need for additional clinical interventions and reducing healthcare costs. Furthermore, the significant pain reduction in the early postoperative period could lead to better patient satisfaction and reduced reliance on additional analgesics, which may improve adherence to the prescribed postoperative care regimen.

Our study demonstrates that PF diclofenac sodium significantly improves postoperative pain management and accelerates epithelial recovery in patients undergoing SPT-assisted TransPRK compared to pranoprofen. The reduction in pain scores was consistent at all time points (2, 24, 48, and 72 h), with PF diclofenac showing superior efficacy. The observation group experienced lower pain scores at each time interval, with the greatest difference observed at 24–48 h, suggesting more effective early pain control. At 24 h, a greater proportion of patients in the PF diclofenac group reported mild pain (VAS ≤ 3), indicating a faster transition to pain relief. These findings align with existing research highlighting the efficacy of diclofenac for postoperative pain control. Importantly, this study provides novel insights by evaluating both pain reduction and epithelial healing, demonstrating that PF diclofenac not only provides superior analgesia but also promotes faster epithelial recovery (4.05 ± 0.88 days vs. 4.32 ± 0.92 days in the control group). The time to BCL removal was similarly shorter in the observation group, further supporting the benefit of PF diclofenac in postoperative recovery. In contrast to other studies that primarily focus on pain management, our study uniquely assesses both pain and epithelial healing, offering comprehensive evidence of PF diclofenac’s role in optimizing early postoperative recovery without compromising safety or efficacy (Abdel-Radi et al., 2023; Yan et al., 2025).

The safety profile of PF diclofenac was comparable to that of pranoprofen, with no significant differences in adverse events between the two groups. The overall incidence of adverse events was low and did not differ significantly (7.4% in the observation group vs. 11.9% in the control group), with no serious adverse events reported. These findings support the safety of PF diclofenac as a postoperative NSAID for TransPRK patients, further reinforced by the absence of significant differences in corneal haze and inflammatory markers between the two groups. Specifically, no substantial effect on corneal haze severity was observed at 1, 3, or 6 months postoperatively, which is critical for preserving visual outcomes. Additionally, the proportion of eyes with higher-grade corneal fluorescein staining was lower in the observation group at day 3, indicating a potential benefit of PF diclofenac in minimizing epithelial damage. These results suggest that PF diclofenac provides effective pain control while maintaining favorable ocular surface integrity and minimizing the risk of complications such as haze or inflammation (Ho, 2024). Given these findings, PF diclofenac represents a promising alternative to pranoprofen for postoperative care following TransPRK, offering improved patient outcomes without increasing the risk of adverse effects. Further studies with larger sample sizes and long-term follow-up are warranted to confirm these results and better understand the long-term safety profile of PF diclofenac in refractive surgery (Jost et al., 2022; Haque et al., 2025).

Our study supports the findings of previous research on the efficacy of diclofenac for postoperative pain management. Jost et al. (2022) demonstrated that postoperative diclofenac significantly reduces pain in patients undergoing topography-guided transepithelial surface ablation (TSA). This reduction in pain was associated with decreased reliance on additional analgesics. Similarly, our study highlights that PF diclofenac offers superior pain relief compared to pranoprofen, providing early postoperative pain management without the need for additional medications. While Jost et al. focused solely on pain reduction, our study extends their findings by also addressing the positive impact of PF diclofenac on epithelial recovery, emphasizing both analgesic and healing benefits. Wang et al. investigated the use of bandage contact lenses soaked in diclofenac for pain relief after TransPRK. The study found significant pain reduction at 2 h postoperatively and minimal differences in haze or visual acuity between groups. However, unlike Wang et al. (2022), our study employed eye drops as the method of diclofenac delivery, which may offer better patient compliance and a more standardized approach to postoperative care. While Wang et al. used a contact lens, which may require more effort from patients, our approach with eye drops shows no significant difference in haze or adverse events, while offering the added convenience and standardization of eye drop administration. Ben Ephraim Noyman et al. (2024) conducted a network meta-analysis comparing multiple topical NSAIDs for pain management after PRK and found diclofenac to be effective, ranking alongside ketorolac and flurbiprofen. They also noted that diclofenac could cause slight delays in re-epithelization, although its effect was less significant compared to other NSAIDs. Our study concurs with Noyman et al. in demonstrating diclofenac’s analgesic benefits, while further emphasizing that PF diclofenac not only provides superior pain control but also modestly accelerates epithelial recovery compared to pranoprofen, without significantly delaying re-epithelization or haze formation. In contrast to previous studies, our research provides a focused comparison of PF diclofenac to pranoprofen, demonstrating both superior early pain control and modestly faster epithelial recovery. By incorporating standardized postoperative care in a single cohort, we offer a comprehensive evaluation of PF diclofenac’s clinical value, without an increase in adverse events, thereby reinforcing its potential as a preferred postoperative NSAID.

The faster healing observed in the observation group treated with PF diclofenac sodium may be attributed to several mechanistic factors. Diclofenac, a NSAID, works by inhibiting cyclooxygenase (COX) enzymes, particularly COX-2, which are involved in the inflammatory process (Goldstein et al., 2022). By reducing inflammation, diclofenac may enhance the healing process by minimizing the negative impact of postoperative inflammation on epithelial recovery. Moreover, PF formulations, free from preservatives such as benzalkonium chloride, are less likely to cause ocular surface toxicity, which could otherwise delay epithelialization (Iwamoto et al., 2017). Preservative-free diclofenac thus appears to provide effective pain relief while promoting a favorable healing environment. This reduction in local inflammation, coupled with a non-toxic formulation, may contribute to the observed improvement in epithelial healing and faster recovery times in comparison to traditional NSAIDs containing preservatives. In terms of broader clinical application, the study’s results advocate for considering PF diclofenac as a preferred NSAID in the management of pain following TransPRK (Alkadi et al., 2024). This could be especially beneficial in populations with concerns about the preservative components of conventional NSAIDs, offering a safer and more effective option for postoperative care. Given that no significant differences were observed in adverse events between the two groups, PF diclofenac may also present a safer profile in terms of ocular and systemic side effects, making it a suitable choice for patients with sensitive ocular conditions or those at risk of drug-related complications (Betz et al., 2024; Betz et al., 2023). These findings provide valuable evidence supporting the clinical application of PF diclofenac in enhancing postoperative recovery, and further studies are warranted to confirm its long-term safety and efficacy across different surgical techniques and patient populations (Way et al., 2024; Hashemi et al., 2022).

This study has several limitations. As a retrospective cohort study, it is subject to selection bias and residual confounding. Although propensity score matching could have addressed these issues, we opted to preserve the full sample to maintain statistical power, as matching would have reduced the sample size. Given the balanced baseline characteristics and prespecified multivariable adjustments, we believe our approach adequately controlled for confounding factors. Another limitation is the lack of a placebo control group. A placebo control was not feasible due to the retrospective design, limiting our ability to definitively attribute the observed outcomes to the treatments. Additionally, long-term dry-eye assessments and corneal nerve imaging were not included, as they were not systematically available in our database. Pain outcomes are inherently subjective and may be influenced by reporting bias. While objective outcomes like epithelial closure were assessed, mechanistic biomarkers (e.g., tear inflammatory mediators, corneal sensitivity) were not available. Furthermore, safety conclusions are limited by the study’s power to detect rare adverse events. The absence of serious events does not rule out potential risks. Future research should address these limitations through prospective randomized controlled trials (RCTs) with placebo controls. Long-term follow-up is needed to assess the sustained effects of PF diclofenac on corneal healing, visual acuity, and the development of dry eye disease. Advanced corneal nerve imaging could provide valuable insights into the mechanisms of pain relief. Moreover, long-term ocular surface evaluations, particularly regarding NSAID use and meibomian gland dysfunction, should be prioritized. Expanding the study to multiple centers would enhance the generalizability of findings and strengthen the evidence for PF diclofenac as a preferred postoperative NSAID for TransPRK patients.

## 5 Conclusion

In patients undergoing SPT-assisted transepithelial PRK, PF diclofenac was associated with lower postoperative pain scores from 2 to 72 h and a higher proportion achieving mild pain at 24 h compared with pranoprofen. Epithelialization and bandage contact lens removal occurred modestly earlier with PF diclofenac. Corneal haze, inflammatory signs, and adverse events were infrequent and comparable, with no serious adverse events observed.

## Data Availability

The raw data supporting the conclusions of this article will be made available by the authors, without undue reservation.
