# Supplementary material for: Clinical outcomes and safety of preservative-free diclofenac sodium eye drops in SPT-assisted transepithelial photorefractive keratectomy
Source: Front Pharmacol. 2026 Apr 15;17:1781133. doi: 10.3389/fphar.2026.1781133 (PMC13124999; doi:10.3389/fphar.2026.1781133)
Supplement: Supplementary file 1 [file Table1.docx]

Supplementary Table S1. Collinearity diagnostics for covariates included in the multivariable-adjusted models

| Covariate | Tolerance | VIF |
| --- | --- | --- |
| Age | 0.842 | 1.188 |
| Sex | 0.913 | 1.095 |
| Preoperative spherical equivalent | 0.476 | 2.101 |
| Central corneal thickness | 0.521 | 1.919 |
| Mean keratometry | 0.688 | 1.453 |
| Preoperative dry-eye risk/MGD status | 0.801 | 1.248 |
| Ablation depth | 0.431 | 2.32 |

Abbreviations: MGD, meibomian gland dysfunction; VIF, variance inflation factor.

Note: Tolerance and VIF were used to assess multicollinearity among covariates included in the multivariable-adjusted models. In this illustrative example, all VIF values were below 5, indicating no evidence of problematic multicollinearity.
